# Application of luminescence-producing *Mycobacterium tuberculosis* strains for evaluating anti-tuberculosis drug and vaccine efficacy in vitro and in vivo

**DOI:** 10.1186/s12879-026-13140-w

**Published:** 2026-04-11

**Authors:** Soo-Min Kim, Eunkyung Shin, Jin-Seung Yun, Jaeseon Jeon, Sohee Park, Yong Woo Jung, Dokeun Kim, Hye-Sook Jeong

**Affiliations:** 1 Division of Infectious Disease Vaccine Research, Center for Vaccine Research, National Institute of Health, Korea Disease Control and Prevention Agency, 212, Osongsaengmyeong 2-ro, Osong-eup, Heungdeok-gu, Cheongju, Chungbuk 28160 Republic of Korea; 2 College of Pharmacy, Korea University, Sejong, 30019 Republic of Korea

**Keywords:** Bioluminescence, Drug development, Growth rate, Tuberculosis, Vaccine efficacy

## Abstract

**Background:**

Tuberculosis (TB), caused by *Mycobacterium tuberculosis* (*M.tb*), remains a major public health problem worldwide. The development of effective drugs and vaccines remains crucial but the prolonged culture period of *M.tb* hampers this process. This limitation has been addressed by developing a bioluminescent *M.tb* strain that can be used to measure bacterial burden in live animals non-invasively and in real time. We investigated whether bioluminescence real-time imaging technologies could be used to monitor the infection process in live mice, with the aim of facilitating TB research.

**Methods:**

In this study, we constructed bioluminescence-expressing *M.tb* strains. We compared the growth of these recombinant reporter and wild-type (WT) strains to confirm their stability in vitro. BALB/c mice were infected via intranasal or intravenous routes and monitored using an In Vivo Imaging System. The efficacy of isoniazid treatment was assessed using bioluminescence imaging and confirmed using colony-forming unit (CFU) assays. In a separate experiment, BCG-vaccinated and naïve control mice were infected with *M.tb* L5-Lux, a recombinant strain that expresses luxCDABE under the L5 promoter derived from the L5 mycobacteriophage. Vaccine efficacy was assessed by comparing bioluminescence and CFU counts.

**Results:**

The *M.tb* L5-Lux strain showed stable growth and a strong bioluminescent signal comparable to the WT strain. In both infection models, bioluminescence intensity strongly correlated with bacterial load. Isoniazid treatment led to a significant reduction in luminescence and CFU. In the vaccine study, BCG-vaccinated mice exhibited lower in vivo and ex vivo bioluminescence and approximately 0.5 log₁₀ lower CFU counts than naïve controls, demonstrating the utility of the *M.tb* L5-Lux strain for vaccine efficacy assessment.

**Conclusions:**

The *M.tb* L5-Lux bioluminescent reporter strain provides a sensitive and efficient tool for monitoring *M.tb* infection and evaluating anti-TB drug and vaccine efficacy in real time. This imaging-based platform facilitates faster and more ethical preclinical testing by reducing the number of animals and experimental duration.

**Supplementary Information:**

The online version contains supplementary material available at 10.1186/s12879-026-13140-w.

## Background

Tuberculosis (TB) is the deadliest bacterial infection globally, with an estimated 10.6 million new cases and 1.3 million deaths in 2022; more than 95% of these fatalities occur in low- and middle-income countries [1]. Although the 6-month multidrug regimen of rifampicin, isoniazid (INH), and pyrazinamide is effective for most drug-susceptible tuberculosis, treatment adherence remains challenging due to prolonged treatment durations, a high pill burden, drug-drug interactions, and adverse drug reactions, such as hepatotoxicity [2]. The rise of multidrug-resistant and extensively drug-resistant strains reduces treatment success and the century-old Bacillus Calmette–Guérin (BCG) vaccine confers inconsistent protection against adult pulmonary TB [3]. These shortcomings underscore the need for new small-molecule drugs, sterilizing combinations, and more efficacious vaccines.

Several intrinsic properties of *Mycobacterium tuberculosis* (*M.tb*) result in the slow preclinical discovery of treatments. First, its lipid-rich cell envelope—composed of mycolic acids, arabinogalactan, and peptidoglycan—acts as a permeability barrier and hosts efflux pumps, providing innate antibiotic tolerance [4]. Second, *M.tb* divides slowly, with generation times of 20–24 h in standard media, and all infection experiments must be performed in Biosafety Level 3 (BSL-3) laboratories, raising infrastructure and labor costs. Conventional efficacy readouts rely on enumerating colony-forming units (CFU); however, lung or spleen homogenates must be incubated for at least 3 weeks before colonies stabilize, creating a time constraint. Moreover, serial sacrifice experiments have ethical and financial burdens [5]. To overcome these limitations, bioluminescence imaging (BLI) using *M.tb* strains expressing luciferase has emerged as a valuable tool for TB research. Using this non-invasive technique, monitoring the bacterial burden in real time in live animals is possible, reducing animal sacrifice and allowing a longitudinal assessment of treatment responses and infection burdens [6]. Thus, BLI may be a faster and more ethical alternative to conventional CFU-based methods. Furthermore, promoter-driven reporter strains, such as L5-Lux, which provide insights into bacterial gene expression during infection, can be used for efficacy evaluation and mechanistic analysis [7].

Traditional methods for assessing bacterial burden, such as counting CFUs, require invasive sampling and prolonged culture periods, resulting in long wait times (e.g., weeks) for results. Optical reporter technologies can circumvent these delays by enabling the non-destructive, real-time quantification of bacterial loads in living animals. Fluorescent proteins, such as GFP and mCherry, provide single-cell resolution in vitro but suffer from tissue autofluorescence, scattering, and limited penetration (< 1 mm in lung parenchyma), rendering whole mouse quantification unreliable [8, 9]. Substrate-dependent luciferases—including firefly luciferase, NanoLuc, Renilla, and Gaussia—offer excellent signal-to-noise ratios and early correlation with CFU [6]. However, each imaging session requires intraperitoneal or intravenous (IV) substrate injections (d-luciferin, furimazine, and coelenterazine), which elevate animal stress, confound longitudinal readouts, and expose staff to sharps and aerosols under BSL-3 containment [10]. Substrate diffusion is also inconsistent in hypoxic, necrotic lesions in chronic tuberculosis, largely due to poor vascularization and a dense caseous structure. These pathological features hinder the effective delivery of small molecules—including imaging substrates and antibiotics—to the central regions of granulomas, as demonstrated in histological and imaging studies [11].

Bacterial lux operons (luxCDABE or luxABCDE) encode luciferase (LuxAB) and reductase enzymes (LuxCDE) that synthesize the long-chain aldehyde substrate required for the luciferase reaction intracellularly. Moreover, endogenous flavin reductases supply reduced flavin mononucleotide (FMNH₂). Light is therefore produced continuously without exogenous reagents—a property termed autobioluminescence. Early lux implementations in *M. smegmatis* and *M.tb* established feasibility but yielded weak signals or unstable plasmids [12]. A robust substrate-free reporter would enable the measurement of photon flux weekly over the course of several months and reduce the size of the animal samples. This is in keeping with the principles of Replacement, Reduction, and Refinement (3Rs). In addition, it would eliminate substrate injections and provide reliable readouts even in hypoxic tissues [13, 14]. Lux based assays in microplates can also deliver minimum-inhibitory-concentration values within 4–6 h compared with 7 days for CFU-based methods [15].

Previously, we engineered an autobioluminescent *M.tb* reporter that produces a bright, stable signal but retains wild-type growth and virulence [7]. Four expression cassettes were constructed by pairing the strong mycobacterial Hsp60 promoter [16] or mycobacteriophage L5 promoter [17] with a codon-optimized luxABCDE operon from *Photorhabdus luminescens*, with or without the co-expression of an FMN reductase (*frp*) to enhance FMNH₂ supply. A single copy of each cassette was integrated at the chromosomal *attB* site of *M.tb* H37Rv. Comparative screening identified the L5-driven construct (hereafter L5-Lux) as ≥ 10-fold brighter than the next best variant while preserving parental growth kinetics and genetic stability [18].

In this study, we characterized L5-Lux in vitro, correlated in vivo photon flux with pulmonary *M.tb* CFUs in intranasal (IN) and IV mouse models, and demonstrated the versatility of this reporter by quantifying the bactericidal action of INH and protective effect of BCG vaccination. Chemotherapy and vaccination represent distinct but complementary pillars of TB control, yet both ultimately depend on reliable, quantitative measurements of mycobacterial burden. By applying the same L5-Lux/IVIS bioluminescence readout and comparing it with conventional CFU enumeration in both drug- and vaccine-intervention experiments, we aimed to validate this strain as a single, versatile imaging platform rather than to directly compare the mechanisms or magnitudes of protection between these interventions. By embedding a substrate-free optical reporter into standard murine TB protocols, we hope to present a rapid, quantitative, and humane platform that streamlines drug discovery and vaccine development for a pathogen long hindered by preclinical bottlenecks.

## Methods

### Bacterial strains and culture conditions

*Escherichia coli* DH5α (Thermo Fisher Scientific, Waltham, MA, USA) was grown at 37 °C in Luria–Bertani (LB) broth (Difco, Detroit, MI, USA) broth or on LB agar plates supplemented with 50 µg/mL kanamycin. *M.tb* strain H37Rv was cultured in Middlebrook 7H9 broth (Difco, Detroit, MI, USA) supplemented with 10% albumin–dextrose–catalase (ADC; Difco, Detroit, MI, USA), 0.5% glycerol (Thermo Fisher Scientific), and 0.05% Tween 80 (Thermo Fisher Scientific). For solid culture, Middlebrook 7H10 agar supplemented with 10% ADC and 0.2% glycerol was used. Incubations were performed at 37 °C in a shaking incubator for broth cultures and humidified chamber for agar plates. Kanamycin was used at 50 µg/mL for *E. coli* and 25 µg/mL for *M.tb*.

### Electroporation of *M.tb* H37Rv

For the preparation of electrocompetent mycobacteria, mid-log growth-phase *M.tb* was incubated on ice for 2 h and harvested by centrifugation at 2700 g and 4℃ for 20 min, after which it was re-suspended and washed using ice-cold phosphate-buffered saline (PBS) containing 10% glycerol three times. The washed cells were re-suspended in ice-cold PBS containing 10% glycerol and 100-µL aliquots were stored at -80℃ before electroporation (40 µF, 2.5 kV, 200 Ω) using a Gene PulserR (BioRad, Hercules, CA, USA).

### Measurement of optical density and bioluminescence

For in vitro bioluminescence analyses, *M.tb* reporter strains (Hsp60-Lux, Hsp60-Lux-frp, L5-Lux, and L5-Lux-frp) were cultured in Middlebrook 7H9 broth supplemented with 0.05% Tween-80 and kanamycin (25 µg/mL) at 37 °C with shaking. Cultures were maintained for up to 45 days. At designated time points (days 0, 10, 20, and 45), 200 µL aliquots of the cultures were transferred to black 96-well optical-bottom plates, and bioluminescence was measured using a multimode microplate reader (BMG Labtech, Ortenberg, Germany). Optical density at 600 nm (OD_600_) was determined using the same instrument to normalize luminescence output. Relative light units (RLU) were divided by OD_600_, and values were log-transformed for comparative analyses.

### Generation of recombinant strains expressing luminescence

To construct recombinant strains expressing bioluminescence via the lux operon, we used the cloning vectors, plasmids, promoters, and reporter genes listed in Additional file 1. The reporter genes sequences were codon-optimized to induce effective expression in *M.tb* H37Rv and were synthesized by GenScript. The optimized fragments were assembled into full-length inserts using gene assembly PCR, and construct integrity was confirmed by Sanger sequencing prior to cloning. These sequences, including promoters and restriction enzyme sites (NotI–EcoRI), were synthesized and inserted into the *E. coli*-*Mycobacterium* shuttle vector pMV306, which included a site for integration into the mycobacterial chromosome [7]. pMV306 with the lux operon (GenBank no. AF4030784.1, *P. luminescens* lux operon) inserted was transformed into *E. coli* (DH5α). Restriction enzyme digestion was used to confirm the presence of the gene fragment. The full-length lux operon was supplied as a sequence-verified synthetic insert, and the integrity of the luxCDABE coding sequence and cloning junctions in the final pMV306-lux construct was further confirmed by Sanger sequencing. The verified vectors were transfected into prepared electrocompetent *M.tb* H37Rv using an electroporator as previously described (section: Electroporation of *M.tb* H37Rv).

Hsp60 or L5 promoters were located in front of luxAB to express the luciferase enzyme. The lux operon comprised a promoter/luxAB (conferring enzyme activity)/pG13 (to produce substrate continuously) [19]/luxCDE (providing substrate). Each designed reporter gene was codon-optimized and the genes were synthesized to include restriction enzyme sites NotI–XbaI for the insertion of the sequence into the multiple cloning site of the integration vector pMV306. All reporter genes, except for the lux operon, were amplified using PCR and the primers and templates listed in Additional file 1. The sequence of the PCR products was confirmed by DNA sequencing.

Recombinant mycobacterial strains were generated by electroporating *M.tb* H37Rv with plasmids containing the designed reporter genes using a 0.2-cm gap cuvette at 2.5 kV, 40 µF, and 200 Ω (single pulse), as previously described [14]. Strains were cultured on Middlebrook 7H10 agar supplemented with oleic acid–albumin–dextrose–catalase (OADC) and kanamycin (25 µg/mL) at 37 °C for 3–4 weeks until colonies were visible. Images of bioluminescent colonies were obtained using an In Vivo Imaging System (IVIS; PerkinElmer, Waltham, MA, USA) with an open emission filter and standard acquisition settings (exposure time: 5 min, binning: medium). Recombinant strains were named according to the plasmid backbone and promoter used to drive lux operon expression (e.g., Hsp60-Lux and L5-Lux).

### Strains and growth conditions

To assess the stability and growth of the recombinant strains, optical density (OD₆₀₀) and CFUs were measured. Reporter strains carrying the lux operon were cultured in 7H9 broth and plated on 7H10 agar (Difco, Detroit, MI, USA) with or without kanamycin. WT and recombinant strains were initially cultured in antibiotic-free medium, followed by CFU counting on kanamycin-supplemented 7H10 agar to confirm plasmid retention and strain stability. Luminescence was also recorded to evaluate reporter gene expression.

Bacterial growth was assessed in 7H9 broth containing 0.5% glycerol. OD₆₀₀ values were measured using a spectrophotometer (BMG Labtech, Ortenberg, Germany). For luminescence assays, *M.tb* reporter strains (inoculum OD₆₀₀ = 0.1) were cultured in minimal medium with 0.5% glycerol (Sigma–Aldrich, St. Louis, MO, USA) in a 5% CO₂ incubator. At the indicated time points (days 0, 10, 20, and 45), 200-µL samples were transferred to black optical-bottom 96-well plates and bioluminescence was measured using a multimode microplate reader.

Infection stocks were prepared from mid-log-phase *M.tb* cultures. For CFU counting, serial 10-fold dilutions (10^− 4^, 10^− 5^, and 10^− 6^) were performed in PBS containing 0.05% Tween 80 (Sigma–Aldrich) and samples were plated onto Middlebrook 7H10 agar. Plates were incubated at 37 °C for 4–5 weeks before CFU counting. A total of five mice were included in each experimental group (*n* = 5), yielding five lung homogenates per group. Each homogenate was plated in triplicate for CFU enumeration, generating three technical replicates per biological sample.

### In vitro validation of viability-linked bioluminescence and INH susceptibility

To confirm that bioluminescence in the *M.tb* L5-Lux strain reflects bacterial viability, cultures were subjected to chemical stress using H₂O₂ and sodium dodecyl sulfate (SDS). Mid-log phase cultures (OD₆₀₀ ≈ 0.3–0.7) were exposed to 3% H₂O₂ or 1% SDS and incubated at 37 °C with shaking. At 0, 3, and 7 days post-exposure, OD₆₀₀ and relative light units (RLU) were measured to monitor bacterial growth and reporter activity. A concurrent decrease in OD and RLU was used to verify that reporter expression was specific to viable bacteria.

To evaluate the susceptibility to INH in vitro, *M.tb* L5-Lux cultures were treated with five concentrations of INH (80, 40, 20, 10, and 0 ng/mL) in two-fold serial dilutions. After 4 h of incubation at 37 °C, OD₆₀₀ and RLU were measured. The lowest concentration that prevented an increase in both parameters was considered indicative of bacterial growth inhibition and metabolic suppression.

### Animals and *M.tb* infection

All animal experiments were conducted in the Animal Biosafety Level 3 (ABSL-3) facility at the Korea National Institute of Health under specific pathogen-free conditions. Mice were housed in individually ventilated cages with a 12-h light/dark cycle, maintained at 22 ± 1 °C and 55 ± 5% humidity. Animals had ad libitum access to sterile water and a standard laboratory rodent diet (LabDiet, PMI Nutrition International, USA). For IVIS imaging studies, animals were switched to an alfalfa-free diet to minimize autofluorescence (Teklad Global 18% Protein Rodent Diet, Alfalfa-Free, Envigo, USA).

Two infection routes were used to model different aspects of TB pathogenesis: IV and IN. For systemic infection modeling, 5 × 10⁷ CFUs in 100 µL of PBS were administered via tail vein injection. For localized pulmonary infection, 5 × 10⁷ CFU in 20 µL of PBS were delivered intranasally under light anesthesia.

At designated time points, mice were euthanized using CO₂ inhalation, followed by lung harvest and homogenization. Each lung was homogenized in 3 mL of sterile PBS. For the IV infection model, lungs were collected on days 1, 21, 35, 49, and 56 post-infection. For the IN model, lungs were harvested on days 1, 21, 28, 35, 42, and 49 post-infection. Bacterial burden was assessed by plating serial 10-fold dilutions (10^− 4^, 10^− 5^, and 10^− 6^) of lung homogenates onto Middlebrook 7H10 agar supplemented with 10% OADC. Plates were incubated at 37 °C for 3–4 weeks and CFUs were counted to quantify pulmonary infection levels. For histopathological analysis, tissue specimens were initially fixed in 10% neutral-buffered formalin, embedded in paraffin, sectioned into 4–5-µm slices, stained with hematoxylin and eosin, and evaluated under a microscope.

### Quantification of cytokines

Blood samples (200–250 µl) were collected and serum was prepared by centrifugation according to standard procedures by allowing clotting at room temperature for 30 min, followed by centrifugation at 13,200 × *g* for 10 min at 4 °C. The resulting supernatants were diluted 1:2 with assay buffer provided in the MIP10-MAG kit (Merck Millipore, Burlington, MA, USA), as per the manufacturer’s protocol. IP-10 (CXCL10) concentrations were measured using the Mouse IP-10 Single-Analyte Magnetic Bead Kit (MIP10-MAG) combined with the multiplex backbone Mouse Cytokine/Chemokine Magnetic Bead Panel (CCYTOMAG-90 K; Merck Millipore). The IP-10-specific bead region supplied in MIP10-MAG was spiked into the CCYTOMAG-90 K bead mix and only this region was analyzed. Diluted samples, standards, and blanks were incubated overnight (16–18 h, 4 °C) with the bead mixture. After incubation, magnetic beads were washed twice using a Bio-Plex Pro Wash Station (Bio-Rad, Hercules, CA, USA) to remove unbound components. After automated washes, a biotinylated anti-mouse IP-10 detection antibody (included in the MIP10-MAG kit; Merck Millipore) and streptavidin-phycoerythrin were added sequentially according to the manufacturer’s instructions. Beads were analyzed on a Luminex 200 instrument (Luminex, Austin, TX, USA). Median fluorescence intensity specific to the IP-10 bead region was recorded using xPONENT (v 4.1.308.0) and converted to pg/mL via a five-parameter logistic standard curve generated in Master Plex QT 2010 (MiraiBio, Hitachi, CA, USA).

### Evaluation of therapeutic and vaccine efficacy

To assess drug efficacy, mice were infected with *M.tb* L5-Lux IN or IV. Beginning 21 days post-infection, animals received 25 mg/kg INH (Sigma-Aldrich, St. Louis, MO, USA) by oral gavage for 3 to 4 weeks (5 days per week). For vaccine studies, mice were immunized subcutaneously with 1 × 10⁶ CFU of *Mycobacterium bovis* BCG (Pasteur strain) 6 weeks before an IN challenge with 5 × 10⁷ CFU of *M.tb* L5-Lux. Efficacy was quantified using longitudinal in vivo bioluminescence imaging performed with an IVIS Spectrum system (PerkinElmer) at multiple time points post-infection. For ex vivo imaging, lungs were harvested and imaged immediately to measure total photon flux. Bacterial burden was further assessed by homogenizing lungs collected at the study endpoint and plating serial dilutions onto Middlebrook 7H10 agar for CFU enumeration.

### Imaging of animals and organs

All in vivo and ex vivo imaging procedures were conducted in an ABSL-3 containment facility in accordance with institutional biosafety protocols. For imaging, mice were anesthetized via the intraperitoneal injection of a ketamine (100 mg/kg) and xylazine (10 mg/kg) mixture. Anesthetized mice were placed in the imaging chamber of the IVIS Spectrum system (PerkinElmer, Waltham, MA, USA) and whole-body bioluminescence images were acquired.

Immediately after in vivo imaging, mice were euthanized using CO_2_ inhalation, and the lungs were excised for ex vivo imaging. Lung tissues (measuring approximately 2 cm) were placed in sterile 24-well plates and imaged using the automatic acquisition mode of the IVIS Spectrum system (PerkinElmer), which adjusts exposure time and binning based on signal intensity. For both in vivo and ex vivo imaging, the same acquisition protocol and instrument settings were applied to all groups within a given experiment to ensure procedural consistency. Following imaging, the lungs were homogenized in 3 mL of PBS and serial 10-fold dilutions (10^− 4^, 10^− 5^, and 10^− 6^) were obtained for CFU determination on Middlebrook 7H10 agar plates.

Bioluminescence signals were quantified using Living Image v4.7.4 (PerkinElmer). Regions of interest (ROIs) were drawn to encompass the entire thoracic area for in vivo imaging or the whole excised lung for ex vivo imaging, using consistent anatomical landmarks, and the same ROI template was copied and applied across time points and mice to standardize ROI size and placement. Signal intensity was expressed as radiance (photons/s/cm²/sr), and background signal was subtracted using ROIs from uninfected control animals. Images within each experiment were acquired in automatic exposure mode (open emission filter) on the IVIS Spectrum system and displayed using a common radiance scale, allowing internally consistent visualization and comparison of luminescence across animals and time points.

### Data analysis

Statistical analyses were performed using GraphPad Prism version 5 (GraphPad Software, San Diego, CA, USA). Bioluminescence data, expressed as total photon flux (photons/s/cm²/sr), and CFU counts were log-transformed prior to statistical testing to normalize distributions. Data are presented as the mean ± standard deviation (SD) of biological replicates, unless otherwise stated. For CFU, the value for each mouse represents the median obtained from triplicate plating of each lung homogenate.

Bioluminescence signal intensity was quantified using the ROI tool in Living Image v4.7.4 (PerkinElmer) and the background signal was subtracted using values obtained from uninfected control animals. All images were acquired using the automatic acquisition mode of the IVIS Spectrum system (PerkinElmer), which automatically adjusts exposure time and binning based on signal intensity. To enable comparisons, the same automatic settings were applied consistently across all groups.

Group comparisons were conducted using unpaired two-tailed Student’s *t-test*s for two-group comparisons or one-way ANOVA followed by post hoc Tukey’s multiple comparisons test for datasets involving more than two groups. Data normality and homogeneity of variance were verified using the Shapiro–Wilk and Brown–Forsythe tests, respectively (*p* > 0.05 for all datasets). Mean differences (Δlog₁₀ CFU) and 95% confidence intervals (CIs) were calculated where appropriate. No data were excluded from the analysis. A *p*-value of less than 0.05 was considered statistically significant.

## Results

### Selection and stability assessment of the brightest bioluminescent recombinant *M.tb* reporter strain in vitro

To identify the most stable and brightest recombinant *M.tb* strain, we compared the luminescence profiles of four reporter constructs: Hsp60-Lux, Hsp60-Lux-frp, L5-Lux, and L5-Lux-frp. The L5-Lux strain consistently exhibited the highest and most stable luminescence throughout the observation period.

**Fig. 1:**
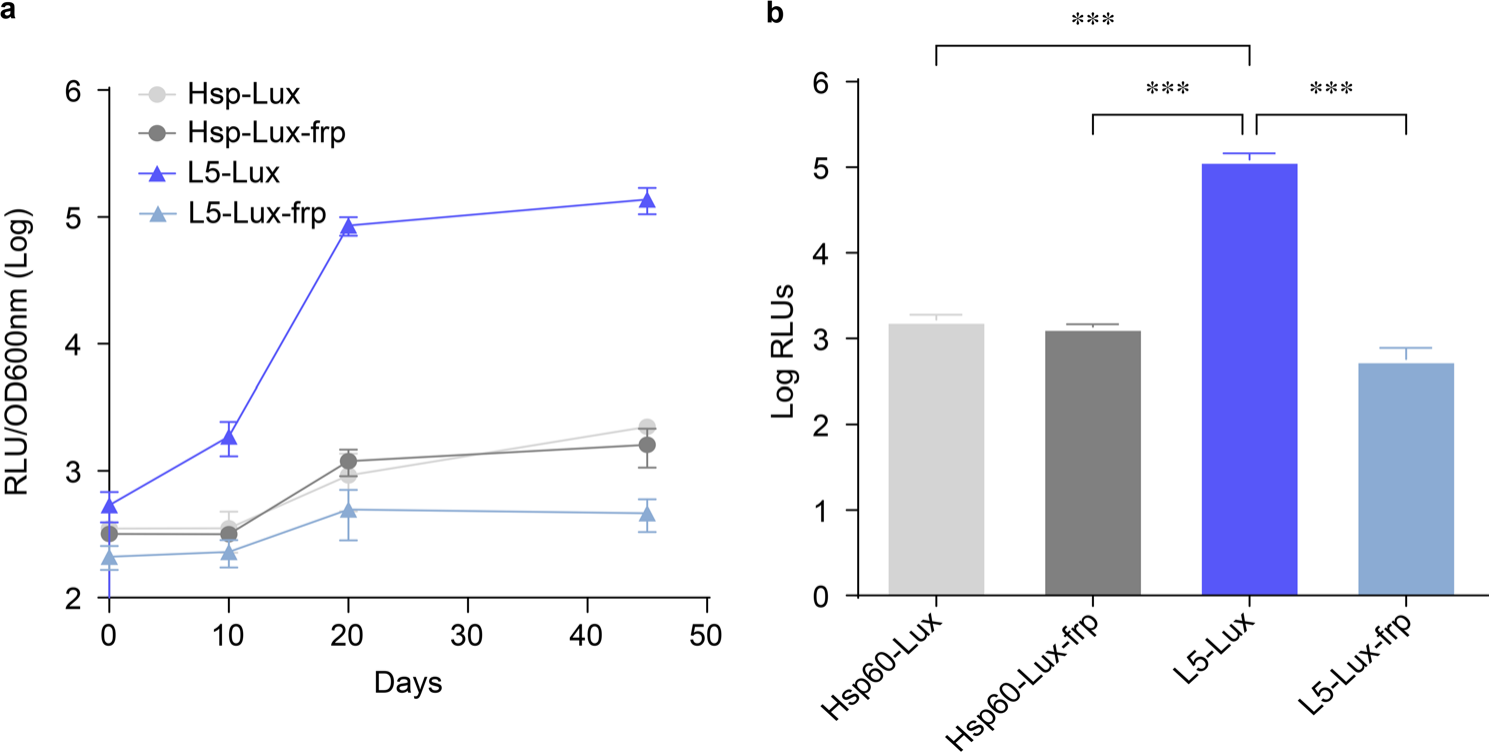
Assessment of the in vitro stability of recombinant *M.tb* bioluminescent reporter strains. (**a**) Bioluminescence intensity was measured during in vitro culture in kanamycin-containing 7H9 medium using recombinant *M.tb* strains engineered to express lux genes under the control of Hsp60 or L5 promoters, with or without the flavin reductase frp. Luminescence values (RLU) were normalized to OD₆₀₀ to account for bacterial growth, and monitored over time (Days 0, 10, 20, 30, and 50) to assess strain stability. Data are shown as mean ± SEM from three independent experiments. (**b**) Endpoint luminescence (Day 50) of each recombinant strain. Statistical significance was evaluated by one-way ANOVA followed by Dunn’s multiple comparison test. *p < 0.001. Data represent the mean ± SD from three independent experiments

All four strains showed stable growth and sustained bioluminescence expression without significant inhibition of bacterial proliferation (Fig. 1a), suggesting that the presence of the lux operon and associated genes did not affect bacterial growth substantially under the tested conditions. Among the strains, the L5-Lux reporter exhibited the strongest luminescence signal throughout the observation period. The *M.tb* L5-Lux strain lacking the *frp* gene produced significantly stronger bioluminescence than its frp-coexpressing counterpart (*p* < 0.001, one-way ANOVA with Tukey’s post-hoc test), contrary to the initial hypothesis that frp would enhance photon output by increasing FMNH₂ availability (Fig. 1b).

**Fig. 2:**
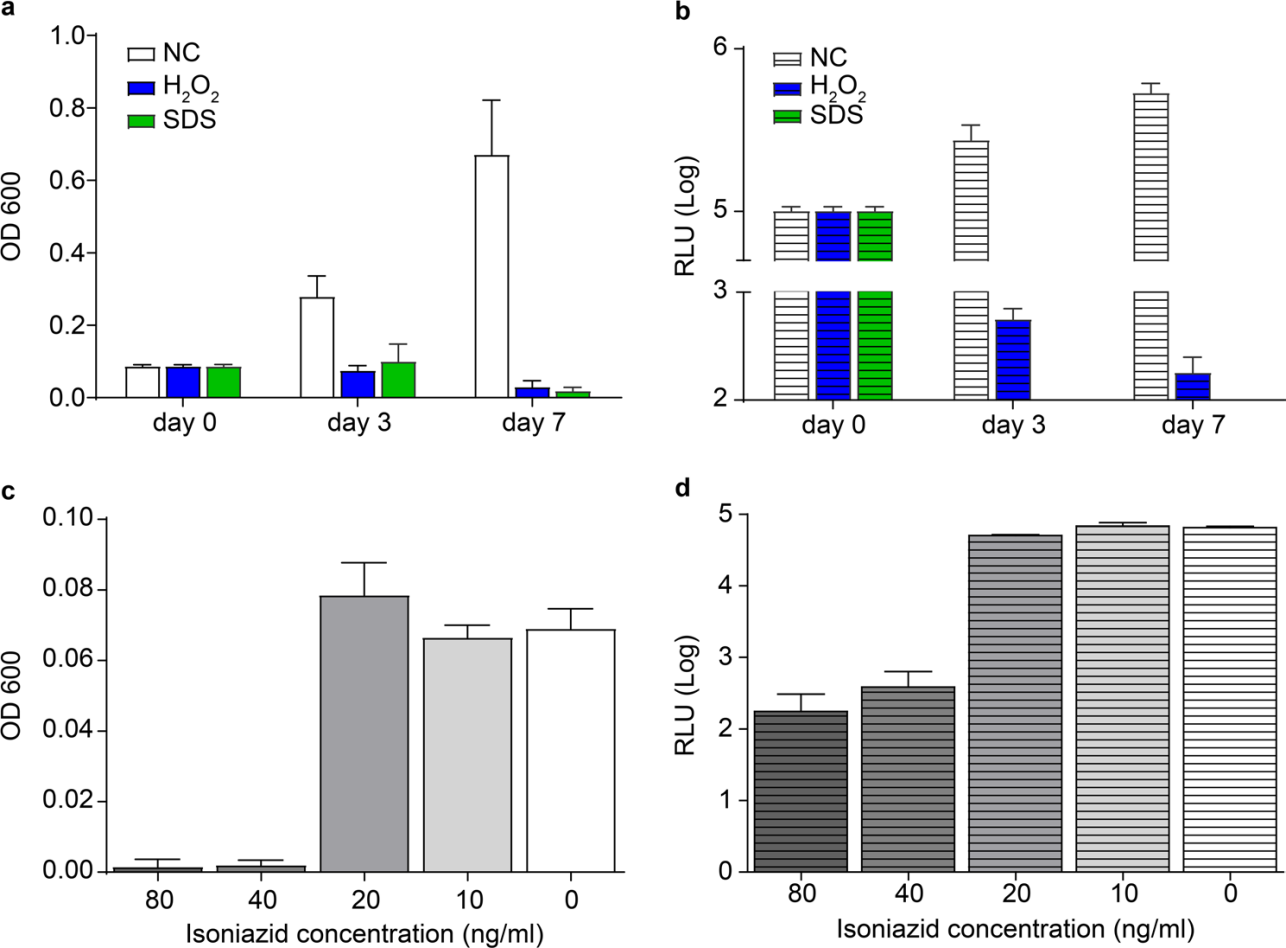
Assessment of stress tolerance and drug susceptibility of the recombinant *M.tb* reporter strain in vitro. (**a**) Growth of the recombinant strain under oxidative (3% H₂O₂) and detergent (1% SDS) stress conditions was evaluated by measuring optical density at 600 nm (OD₆₀₀) on Days 0, 3, and 7. (**b**) Bioluminescence intensity (RLU, log₁₀ scale) was measured in parallel to assess bacterial metabolic activity under the same stress conditions. (**c**) Bacterial growth in response to increasing concentrations of isoniazid (INH) was assessed by OD₆₀₀ measurements after 7 days of incubation. (**d**) Luminescence-based susceptibility testing was performed by measuring RLU values across a range of INH concentrations. **Data represent the mean ± SD from three independent experiments. Statistical analysis was performed using one-way ANOVA followed by Dunn’s multiple comparison test. Significant differences (*p* < 0.001) are indicated by *

**Fig. 3:**
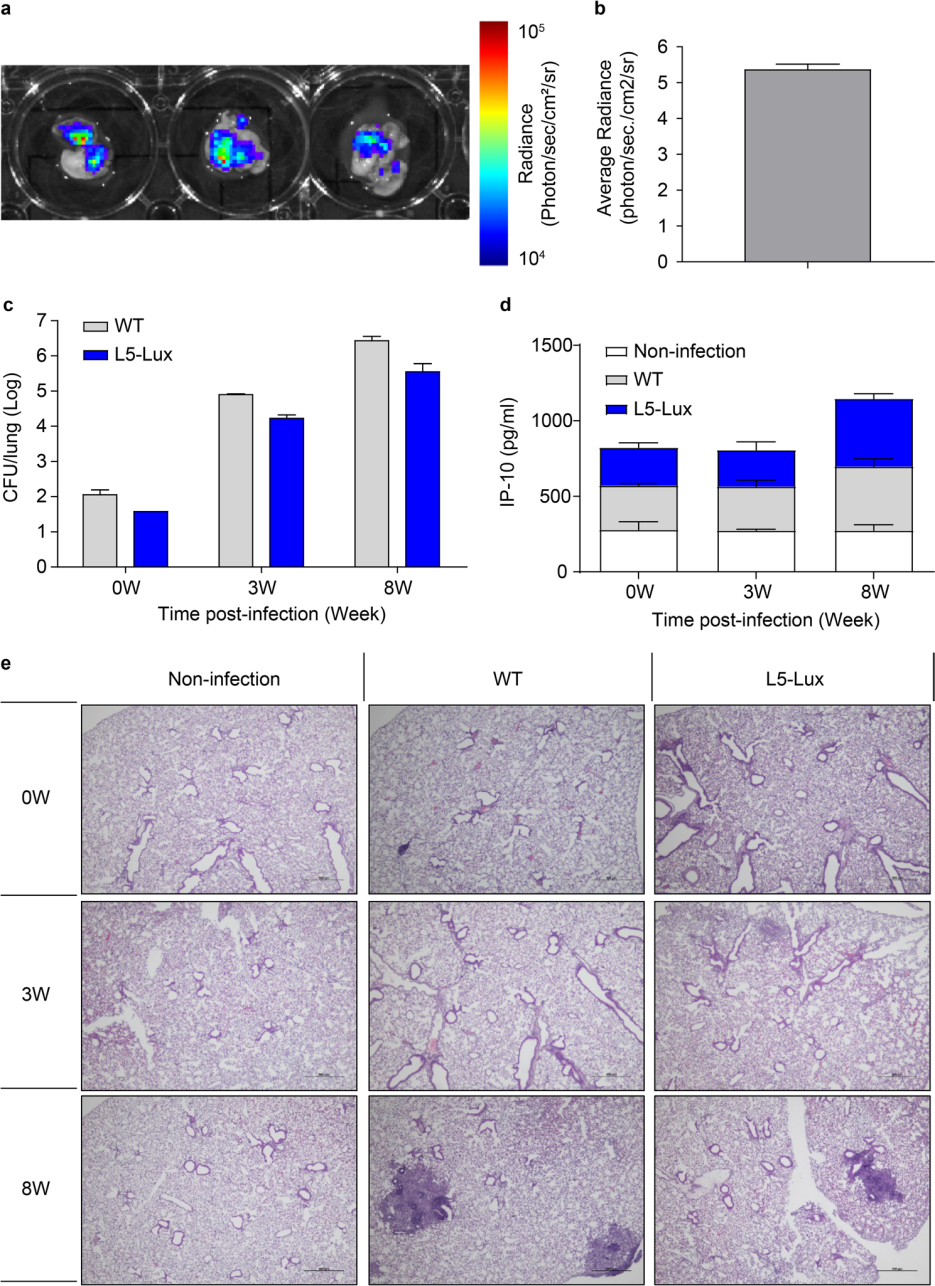
In vivo evaluation of the luminescence expression and pathogenicity of *M.tb* L5-Lux in a mouse model. (**a**) BALB/c mice (*n* = 5 per group) were intranasally infected with *M.tb* L5-Lux, and the luminescence was measured ex vivo using an In Vivo Imaging System (IVIS) at 3 weeks post-infection. (**b**) Quantification of luminescence in the lung was performed by region-of-interest analysis, and is presented as radiance efficiency (photons/s/cm²/sr). (**c**) Bacterial burden in lung tissue was determined by colony-forming unit (CFU) enumeration at 0, 3, and 8 weeks post-infection. (**d**) Levels of interferon gamma-induced protein 10 (IP-10) in lung homogenates were quantified at 0, 3, and 8 weeks post-infection in non-infected, WT, and L5-Lux-infected mice using the Single-Analyte Magnetic Bead Kit. (**e**) Lung pathology was evaluated by hematoxylin and eosin (H&E) staining of lung sections collected at 0, 3, and 8 weeks. Representative images show inflammation and granulomatous lesions in each group. Data are presented as mean ± SD from three independent experiments

**Fig. 4:**
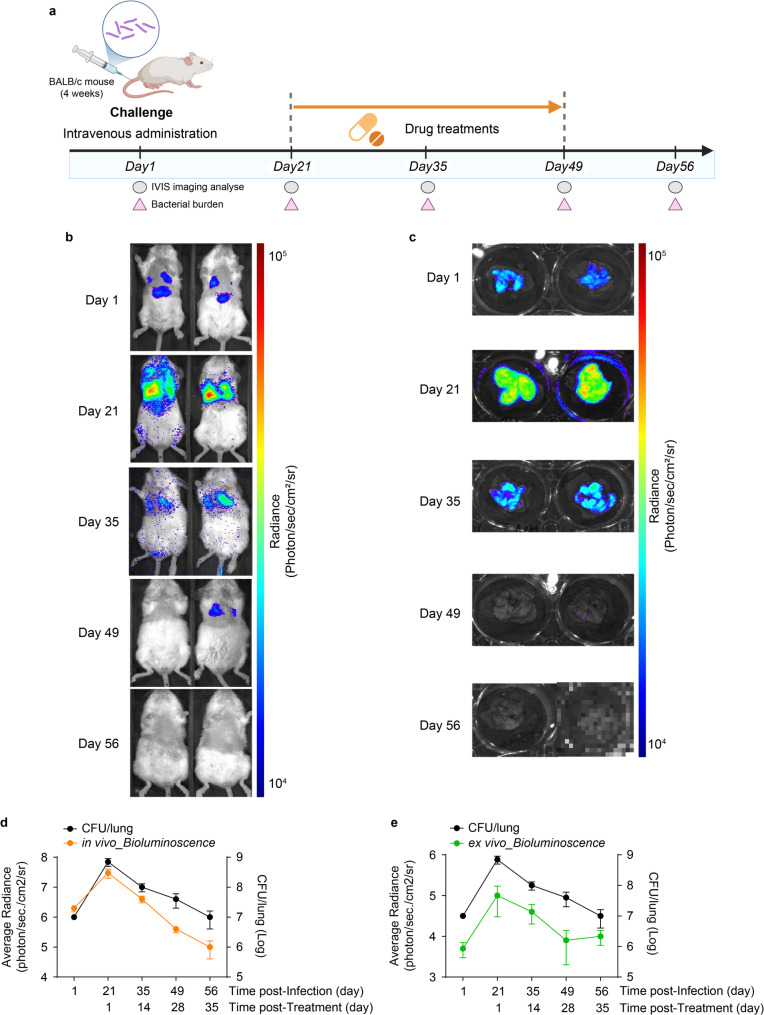
Assessment of anti-tubercular drug efficacy using in vivo and ex vivo bioluminescence imaging in an intravenous *M.tb* L5-Lux infection model. (**a**) Schematic of the experimental timeline. BALB/c mice (*n* = 5 per group) were intravenously infected with *M.tb* L5-Lux and treated with isoniazid (INH) via daily oral gavage from Day 21 to Day 56. In vivo imaging and bacterial burden analysis were performed at the indicated time points (Days 1, 21, 35, 49, and 56 post-infection). (**b**) In vivo bioluminescence images of infected mice were acquired using the IVIS system to monitor infection progression and the response to treatment. (**c**) Ex vivo luminescence images of lungs collected at the same time points. (**d**) Quantification of in vivo luminescence (photons/s/cm²/sr) and lung CFUs over time. (**e**) Quantification of ex vivo luminescence and CFU counts in dissected lung tissues. Luminescence was quantified using region-of-interest (ROI) analysis. Bacterial burden was determined by plating lung homogenates on 7H10 agar containing kanamycin. Data are presented as mean ± SD from three independent experiments. Data met normality and equal variance assumptions (Shapiro–Wilk and Brown–Forsythe tests, *p* > 0.05), and no samples were excluded

**Fig. 5:**
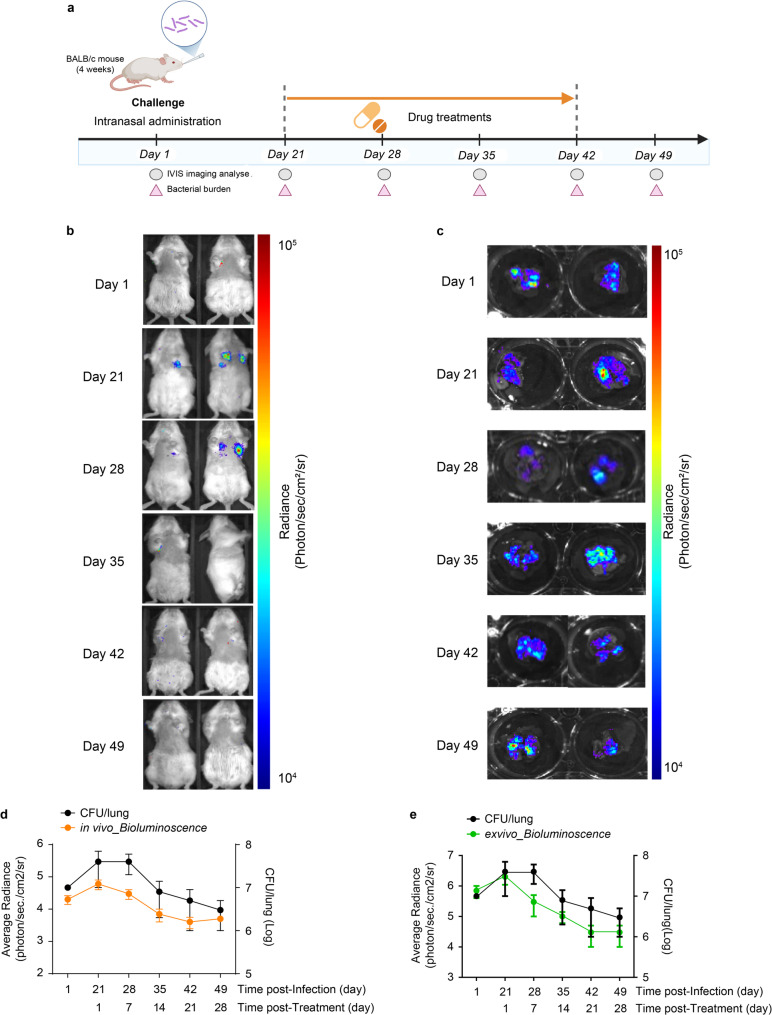
Evaluation of anti-tubercular drug efficacy using bioluminescence imaging in an intranasal *M.tb* L5-Lux infection model. (**a**) Schematic of the experimental design. BALB/c mice (*n* = 5 per group) were intranasally infected with *M.tb* L5-Lux and treated with isoniazid (INH) via daily oral gavage from Day 21 to Day 49. In vivo imaging and bacterial burden analyses were performed at the indicated time points (Days 1, 21, 28, 35, 42, and 49 post-infection). (**b**) In vivo bioluminescence images of mice were acquired using the IVIS system to monitor pulmonary infection and treatment response. (**c**) Ex vivo luminescence images of the lung collected at the indicated time points following in vivo imaging. (**d**) Quantification of in-vivo luminescence intensity (photon flux; photons/s/cm²/sr) and corresponding lung bacterial burden (CFU) for infection and treatment. (**e**) Quantification of ex vivo luminescence signals and CFU counts from dissected lung tissues. Luminescence was measured by region-of-interest (ROI) analysis, and bacterial burden was assessed by plating lung homogenates on 7H10 agar supplemented with kanamycin. Data are presented as mean ± SD from three independent experiments. The data met the Shapiro-Wilk and Brown-Forsythe assumptions (*p* > 0.05), and no samples were excluded

**Fig. 6:**
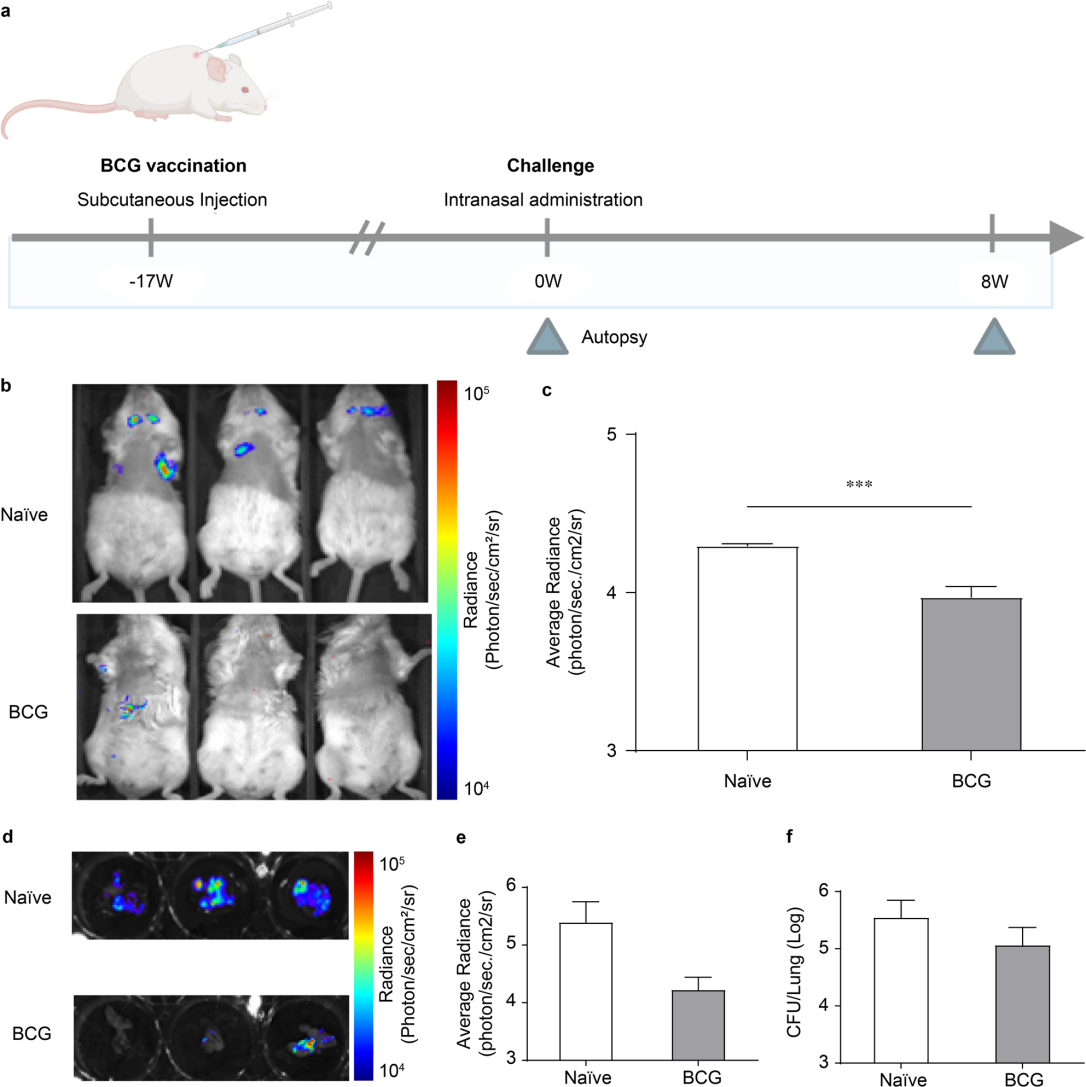
Evaluation of vaccine efficacy in vivo and ex vivo using L5-Lux recombinant strains. (**a**) Schematic of the experimental design. Mice (*n* = 5 per group) were vaccinated with Bacillus Calmette–Guérin (BCG) via subcutaneous injection at Week 17, followed by an intranasal challenge with *M.tb* L5-Lux at Week 0. Lung tissues were collected at 8 weeks post-infection. (**b**) In vivo bioluminescence images of naïve and BCG-vaccinated mice were obtained at 8 weeks post-infection using the IVIS system. (**c**) Quantification of in vivo luminescence intensity (photons/s/cm²/sr) based on region-of-interest (ROI) analysis. (**d**) Ex vivo luminescence images of lungs harvested from the same mice. (**e**) Quantification of ex vivo luminescence from lung tissues. (**f**) Bacterial burden measured as CFU in lung homogenates plated on 7H10 agar supplemented with kanamycin. **Data are presented as mean ± SD from three independent experiments. Statistical significance was determined using a one-way ANOVA followed by Dunn’s multiple comparison test. Significant differences (*p* < 0.001) are indicated by *

Quantitative analysis confirmed that L5-Lux emitted significantly higher bioluminescence than all other constructs (*p* < 0.001), making it the leading candidate for in vivo imaging and efficacy testing. We next evaluated the in vitro stability of the chromosomally integrated lux cassette under both selective and non-selective conditions. WT H37Rv and the four reporter strains were cultured in Middlebrook 7H9 medium without kanamycin and with kanamycin (25 µg/mL) for up to 45 days. Under both conditions, reporter strains displayed optical density and CFU curves that closely paralleled those of WT, indicating that insertion of the pMV306-based lux cassette did not impose a major fitness cost or grossly impair survival over the 45-day period (Additional file 1: Figure S4A, B). Because the kanamycin resistance gene is encoded within the integrated cassette, we also serially passaged L5-Lux in kanamycin-free 7H9 and then plated cultures onto kanamycin-containing 7H10 agar to assess cassette retention. L5-Lux maintained CFU counts comparable to WT (on antibiotic-free plates) and continued to grow on kanamycin-containing plates without evidence of kanamycin-sensitive subpopulations, while retaining a readily detectable bioluminescent signal (Additional file 1: Figure S4C). These findings indicate that the integrated lux cassette is stably maintained during prolonged in vitro culture even in the absence of antibiotic selection and that L5-Lux can be propagated under antibiotic-free conditions prior to in vivo use. Taken together with the intranasal head-to-head benchmarking experiment (Supplementary Figure S3), these data indicate that the L5-Lux reporter strain outperforms our earlier Hsp-Lux construct in terms of luminescence intensity, signal stability, and quantitative correlation with CFU, and we therefore selected L5-Lux as the final autobioluminescent reporter for all subsequent in vivo drug and vaccine efficacy experiments.

### Susceptibility of the recombinant *M.tb* L5-Lux strain to oxidative and detergent stress and INH treatment in vitro

To evaluate the physiological robustness of the *M.tb* L5-Lux reporter strain, we exposed bacterial cultures to oxidative and detergent stress conditions. Cultures were treated with 3% H₂O₂ or 1% SDS and growth (OD₆₀₀) and luminescence (RLU) were measured on days 0, 3, and 7. OD₆₀₀ values were significantly lower in the H₂O₂ and SDS-treated groups than in the untreated control (NC), indicating inhibition of bacterial growth under stress conditions (Fig. 2a). Correspondingly, RLU values also declined over time in both treatment groups, reflecting a reduction in bacterial metabolic activity (Fig. 2b).

To assess the susceptibility of *M.tb* L5-Lux to INH, bacterial cultures were exposed to a two-fold dilution series of INH (0–80 ng/mL) for 4 h. OD₆₀₀ values remained unchanged at concentrations up to 20 ng/mL but were reduced at 40 and 80 ng/mL (Fig. 2c), indicating a threshold-dependent growth inhibition. RLU values showed a similar trend, with no notable decrease up to 20 ng/mL and a substantial reduction at higher concentrations (Fig. 2d). These results demonstrate that the *M.tb* L5-Lux strain reliably reports metabolic inhibition under antibiotic pressure and stress, supporting its utility as an in vitro reporter system for evaluating drug susceptibility and physiological perturbation.

### Validation of recombinant *M.tb* L5-Lux strain infectivity in mice

In an exploratory intranasal dose-optimization experiment, BALB/c mice were challenged with M.tb L5-Lux at 5 × 10⁷, 5 × 10⁸, or 10⁹ CFU, and lung bioluminescence was monitored longitudinally by IVIS imaging (Supplementary Figure S1; Additional file 2). At 20 days post-infection, the 5 × 10⁷ CFU dose provided a robust and relatively homogeneous lung signal with acceptable variability, whereas increasing the inoculum to 5 × 10⁸ or 10⁹ CFU did not substantially enhance signal intensity but raised concerns regarding animal welfare. Based on these pilot data, we selected 5 × 10⁷ CFU as the optimized intranasal inoculum for all subsequent IN infection experiments. To evaluate the infection dynamics of the recombinant *M.tb* L5-Lux strain, mice were infected via the IN route and bacterial burden, bioluminescence, cytokine responses, and histopathological changes were assessed over time. To confirm the stability of the luminescence reporter gene, bioluminescence was visualized in lung tissue 3 weeks after infection using IVIS imaging (Fig. 3a). The intensity of the bioluminescent signal was quantified and expressed as RLU (Fig. 3b). The strong luminescence observed in lung tissues confirmed the successful colonization and persistence of *M.tb* L5-Lux in the murine lungs.

To compare infectivity with the WT strain, lung bacterial burdens were measured 0, 3, and 8 weeks post-infection (Fig. 3c). *M.tb* L5-Lux showed similar replication kinetics to the WT strain, indicating that the lux operon insertion did not impair the ability of the strain to establish pulmonary infection.

To assess host immune responses, serum IP-10 (CXCL10) levels were measured 0, 3, and 8 weeks post-infection. WT- and *M.tb* L5-Lux-infected mice showed higher IP-10 levels than uninfected controls by week 8 (Fig. 3d), suggesting that *M.tb* L5-Lux elicits a Th1-type response comparable to that induced by the WT strain.

Histopathological analysis of lung tissues collected 0, 3, and 8 weeks post-infection and stained with hematoxylin and eosin revealed characteristic granulomatous lesions at 8 weeks in WT- and *M.tb* L5-Lux-infected groups (Fig. 3e). No pathological findings were observed in uninfected controls.

Overall, these results demonstrate that the recombinant *M.tb* L5-Lux strain successfully infects murine lungs, exhibits pathogenic and immunogenic characteristics comparable to the WT strain, and enables the stable bioluminescence-based monitoring of infection.

### Evaluation of therapeutic efficacy across localized pulmonary and systemic infection models

To evaluate route-dependent differences in therapeutic response, we used two distinct murine infection models. IN infection mimics pulmonary tuberculosis, which involves localized infection of the lungs through natural respiratory exposure. In contrast, IV infection induces systemic tuberculosis, characterized by the hematogenous dissemination of bacteria to multiple organs. This dual-model system enables a comparison of drug efficacy in localized and disseminated TB [20]. Bioluminescent imaging using the *M.tb* L5-Lux strain enabled the non-invasive, real-time monitoring of INH activity throughout infection progression. A pilot study was conducted to determine an appropriate intranasal inoculum dose, and 5 × 10^7^ CFUs was selected based on the ability to produce a uniform pulmonary infection with minimal variability (Supplementary Fig. 1).

Intravenous inoculation with 5 × 10⁷ CFUs yielded whole-body luminescence (Fig. 4a). In vivo lung radiance peaked at approximately 3 × 10⁷ photons/s/cm²/sr on day 21 and decreased by approximately 80% after 2 weeks of INH, reaching a plateau by day 56 (Fig. 4b, d). Ex vivo imaging of excised lungs showed a similar decline in photon flux, with bacterial burdens (CFU/lung) decreasing by 1.85 log₁₀ ± 0.22 (95% CI 1.63–2.07, *n* = 5, *p* < 0.01) between days 21 and 56 (Fig. 4c, e).

BALB/c mice were infected intranasally with 5 × 10⁷ CFU of *M.tb* L5-Lux (Fig. 5a), resulting in luminescent signals confined to the thoracic cavity. Thoracic radiance rose to approximately 6 × 10⁴ photons/s/cm²/sr on day 21 and decreased by roughly 50% after 1 week of INH, gradually declining toward baseline by day 49 (Fig. 5b, d). Ex vivo imaging of excised lungs confirmed a comparable reduction in photon flux (Fig. 5c, e) and lung CFUs decreased by 1.13 log₁₀, ± 0.44 (95% CI 0.74–1.51, *n* = 5) between days 21 and 49 post-infection, consistent with the optical signal.

Ex vivo lung imaging and CFU counting at the treatment endpoint showed nearly identical bacterial burdens in IV and IN cohorts (Figs. 4e and 5e), indicating that early route-specific differences in regression kinetics converge once therapy is complete. Together, these results validate the L5-Lux reporter as a robust and flexible tool for the non-invasive quantification of anti-tuberculosis drug efficacy. By enabling a real-time, longitudinal assessment of bacterial dynamics, the model captures transient differences in treatment response between infection routes and provides reliable endpoint data aligned with CFU benchmarks.

### Vaccine efficacy assessment using *M.tb* L5-Lux recombinant strains

To evaluate the utility of the *M.tb* L5-Lux reporter strain for vaccine studies, BALB/c mice were subcutaneously immunized with BCG or left unvaccinated (naïve control), followed 17 weeks later by the intratracheal instillation of *M.tb* L5-Lux. In vivo and ex vivo bioluminescence imaging was performed 0 and 8 weeks post-infection, with the 8-week time point selected to assess protective effects during the chronic phase of infection (Fig. 6a).

At 8 weeks post-infection, unvaccinated mice exhibited strong pulmonary bioluminescence signals. In contrast, BCG-vaccinated mice showed lower in vivo radiance than naïve controls (Fig. 6b, c), indicating that bioluminescent imaging sensitively detects vaccine-induced protection. Ex vivo imaging of lung tissues confirmed a corresponding decrease in luminescence intensity in the BCG group relative to controls (Fig. 6d, e). Quantitative analysis revealed that BCG vaccination resulted in an approximately 0.5 log₁₀ reduction in lung CFUs for WT and *M.tb* L5-Lux strains compared with unvaccinated mice (Fig. 6f), supporting a close alignment between bioluminescent signal reduction and bacterial burden.

These results indicate that BCG immunization provides significant protection against pulmonary *M.tb* L5-Lux infection and that bioluminescent imaging offers a sensitive, non-invasive approach for the rapid evaluation of vaccine efficacy.

## Discussion

This study demonstrates that combining a recombinant *M.tb* L5-Lux strain with an IVIS provides a rapid and quantitative platform for TB research. IVIS shortens observation time and, by enabling longitudinal tracking of the same animals, reduces the number of mice required compared with traditional CFU endpoint assays [21]. We, therefore, set out to verify that this platform can reliably assess infection dynamics, drug efficacy, and vaccine-induced protection.

Luciferase output was optimized by pairing several mycobacterial promoters with a codon-optimized luxCDABE cassette. The mycobacteriophage L5 promoter, recognized by σ^A and σ^B, delivered ≥ 10-fold stronger light signals than the canonical hsp60 promoter with unchanged growth kinetics (Fig. 1) [17, 22, 23]. All recombinant strains displayed growth curves and CFU counts comparable to WT cells and maintained stable autobioluminescence for 50 days. Unexpectedly, co-expression of the flavin-reductase gene frp slightly reduced RLUs under both promoters (Fig. 1). This result was notable because frp was expected to improve luminescence by increasing intracellular FMNH_2_ availability, thereby facilitating the LuxAB-catalyzed light-emitting reaction. Instead, the detected decrease suggests that the overexpression of frp disrupts the cellular redox balance or introduces a metabolic burden [24]. This “frp paradox” may reflect (i) LuxAB inactivation by reactive oxygen species generated during FMNH₂ redox cycling, (ii) competition for NAD(P)H with central metabolism that reduces the reducing power available for bioluminescence, or (iii) the emergence of a new bottleneck at LuxCDE once FMNH₂ is no longer limiting. Similar shifts in rate-limiting steps have been described in bioluminescent systems of Staphylococcus aureus and *Escherichia coli* [25–30]. Future work will test catalytically improved FRP variants or inducible frp expression to resolve this balance. In head-to-head intranasal infections, L5-Lux also generated brighter and more stable bioluminescent signals than Hsp-Lux at matched bacterial burdens (Supplementary Figure S3), reinforcing its suitability as a standardized, substrate-free reporter strain for downstream efficacy studies.

The use of autobioluminescent *M.tb* enabled the real-time, non-invasive monitoring of infection dynamics across two distinct disease models. To establish an optimal inoculum for reliable pulmonary infection, we conducted a pilot study comparing 5 × 10⁷, 5 × 10⁸, and 10⁹ CFUs. Based on criteria used in prior studies [20, 31], 5 × 10^7^ CFUs was selected as it yielded the most uniform infection pattern without excessive nasal retention or variability (Supplementary Fig. 1). To establish an optimal inoculum for reliable pulmonary infection, our pilot study comparing 5 × 10⁷, 5 × 10⁸, and 10⁹ CFUs confirmed that 5 × 10⁷ CFUs yielded the most uniform infection pattern in intranasally challenged mice without excessive nasal retention or variability. For the IV model, we used 5 × 10⁷ CFUs—an inoculum widely reported to generate stable systemic dissemination [32–34]. Pulmonary photon flux correlated linearly with lung CFU and IP-10 upregulation and histopathology were indistinguishable from WT infection, indicating that luciferase insertion does not attenuate virulence [35].

Drug efficacy was assessed using oral INH [36–42]. In IV-infected mice, lung-ROI radiance fell by approximately 80% and lung CFU by 1 log₁₀ within 14 days, whereas IN-infected animals showed a more gradual 50% decline in thoracic radiance over the same interval. However, ex vivo results differed: lung photon flux and CFU converged to nearly identical values irrespective of infection route (Figs. 4 and 5). Thus, IVIS captures early pharmacodynamic differences that terminal endpoints can obscure. Longitudinal imaging provided statistically robust efficacy data in 2 weeks—approximately 80% faster than CFU readouts—and reduced mouse use from approximately 40 to fewer than 15 per compound series, fully meeting the 3Rs principle [7, 43, 44].

Beyond its technical advantages, the model also revealed route-specific kinetic differences. IV infection led to a steeper decline in luminescence during early treatment, likely due to bacterial localization near vascularized lung surfaces, allowing faster drug access via the bloodstream [45]. In contrast, IN infection showed a more gradual reduction in signal, possibly reflecting deeper bacterial seeding within parenchymal lung tissue, where drug penetration is slower [46]. Despite these early differences, ex vivo photon flux and CFU counts converged at the end of treatment. This indicates that effective therapy overcomes route-dependent disparities. Although the magnitude of pulmonary photon flux differed depending on the infection route, effective therapy led to a convergence of photon flux signals and CFU reductions across routes. This convergence underscores the value of this model for real-time monitoring, reduced reliance on endpoint CFUs, and early decision-making in drug development workflows.

Our platform was sensitive to vaccine-induced protection. Eight weeks after BCG immunization, both pulmonary photon flux and lung CFU counts were reduced by approximately 0.5 log₁₀ compared with those in unvaccinated controls, consistent with protective effects reported in other BCG vaccine studies [47, 48]. Because autobioluminescence depends on active bacterial replication, a diminished signal directly reports growth inhibition, offering a rapid, humane alternative to terminal CFU enumeration in early vaccine screening [9].

This study has several limitations. First, we used IN and IV challenges with route-specific INH regimens (4 weeks for the IV model and 3 weeks for the IN model) to accelerate model establishment and to match disease kinetics. Although this internal standardization improved within-model reproducibility, the unequal exposure times and the use of non-aerosol infection routes complicate direct extrapolation to human disease; future work will incorporate aerosol infection and harmonized, exposure-normalized pharmacodynamic endpoints (e.g., AUC of log-transformed signal changes).

Second, longitudinal imaging data were analyzed at the group level (mean ± SD at each time point), which clearly reflected overall treatment trends but did not capture within-animal variability over time or allow reconstruction of per-mouse photon flux–CFU pairs for formal correlation and regression analyses. In addition, the BCG experiment was designed as a small proof-of-concept study, and formal prospective power calculations to detect smaller effect sizes were not performed. In future studies, we plan to collect and retain per-animal longitudinal data, apply mixed-effects models and explicit correlation/regression analyses, and perform a priori power calculations for vaccine efficacy experiments to more rigorously quantify the relationship between bioluminescent signal and bacterial burden.

Finally, lung histopathology in this study was assessed qualitatively to confirm granulomatous changes, and standardized lesion scoring was not performed. Future vaccine experiments will incorporate structured lung pathology scoring alongside CFU and bioluminescence readouts, and, in parallel, integrating AI-based image segmentation and next-generation ilux reporters could further improve sensitivity in hypoxic granulomas and deep tissue sites [27, 49].

## Conclusions

We established *M.tb* L5-Lux bioluminescent imaging as a rapid, sensitive platform for tracking infection, evaluating drug efficacy, and screening vaccine efficacy—providing real-time insights and reducing reliance on CFU assays and animals. By enabling the constant, quantitative monitoring of TB in vivo, this system will serve as a valuable tool to accelerate preclinical TB research and facilitate therapeutic development.

## Supplementary Information

Below is the link to the electronic supplementary material.

Supplementary Material 1: Additional file 1: Supplementary Methods, Tables, and Figures. This file contains (i) a Supplementary Method describing the overlap extension PCR (OE-PCR) strategy and cloning procedures used to construct the luminescent M.tb reporter cassettes, (ii) Supplementary Tables S1 and S2, which summarize the key genetic elements, plasmid constructs, recombinant M.tb reporter strains, and primers used, and (iii) Supplementary Figures S1–S4, which present in vivo and ex vivo bioluminescence imaging of BALB/c mice intranasally infected with different inocula of M.tb L5-Lux over time, intranasal infection and in vivo growth of recombinant M.tb reporter strains, a head-to-head comparison of Hsp-Lux and L5-Lux, and in vitro stability of the luminescent reporter strains under ±kanamycin conditions

Supplementary Material 2: Additional file 2: Raw IVIS and CFU data underlying main and supplementary figures. This excel file containing group-level mean ± SD radiance and CFU values linked to Figures 3–6 and Figure S1

## Data Availability

The datasets used and/or analysed during the current study are available from the corresponding author on reasonable request.

## Abbreviations

BCG: Bacillus Calmette–Guérin
CFU: Colony-forming unit
INH: Isoniazid
IVIS: In Vivo Imaging System
*M.tb*: *Mycobacterium tuberculosis*
TB: Tuberculosis
