# Supplementary material for: Application of luminescence-producing Mycobacterium tuberculosis strains for evaluating anti-tuberculosis drug and vaccine efficacy in vitro and in vivo
Source: BMC Infect Dis. 2026 Apr 11;26:1083. doi: 10.1186/s12879-026-13140-w (PMC13235083; doi:10.1186/s12879-026-13140-w)
Supplement: Supplementary file 1 — Supplementary Material 1: Additional file 1: Supplementary Methods, Tables, and Figures. This file contains (i) a Supplementary Method describing the overlap extension PCR (OE-PCR) strategy and cloning procedures used to construct the luminescent M.tb reporter cassettes, (ii) Supplementary Tables S1 and S2, which summarize the key genetic elements, plasmid constructs, recombinant M.tb reporter strains, and primers used, and (iii) Supplementary Figures S1–S4, which present in vivo and ex vivo bioluminescence imaging of BALB/c mice intranasally infected with different inocula of M.tb L5-Lux over time, intranasal infection and in vivo growth of recombinant M.tb reporter strains, a head-to-head comparison of Hsp-Lux and L5-Lux, and in vitro stability of the luminescent reporter strains under ±kanamycin conditions [file 12879_2026_13140_MOESM1_ESM.docx]

**Additional file 1. Supplementary Method, Tables, and Figures**

**Supplementary Method**

**Overlap Extension PCR (OE-PCR)**

The luxAB, promoters, luxCDE, and frp genes were optimized to improve expression and then individually synthesized using commercial gene synthesis services. For each gene, custom primers were designed wherein overlapping sequences (15–30bp) were incorporated at the ends to enable subsequent fragment fusion via Overlap Extension PCR (OE-PCR). Overlapping primers were designed for each gene segment to ensure complementary annealing with adjacent fragments. The internal primers comprised overlapping regions matching the adjacent gene's termini, while external primers flanked the composite construct. Primer sequences and overlap designs were planned to ensure directional assembly according to the desired gene order. Each gene was separately amplified by PCR using their respective primers and Phusion® High-Fidelity DNA Polymerase (New England Biolabs, Ipswich, MA, USA, M0530). The PCR protocol comprised an initial denaturation at 95°C for 3 min, followed by 25–30 cycles of denaturation (95°C, 30 s), primer annealing (55–65°C, 30 s), extension (72°C, 1 min/kb), and a final extension at 72°C for 5 min. PCR products were purified with a commercial kit (Qiagen, Hilden, Germany, 28104). Purified gene fragments were mixed at equimolar concentrations for OE-PCR without additional primers. The reaction conditions mirrored those above, except that cycling was reduced to 10–15 cycles to allow extension across overlaps. Resulting products were amplified in a subsequent PCR using outermost primers, with standard cycling conditions (20–25 cycles), yielding the full-length assembled gene. The assembled gene construct was ligated into a shuttle vector. Recombinant plasmids were screened by restriction enzyme digestion using NotI (Thermo Fisher Scientific, Waltham, MA, USA, FD0595) and XbaI (Thermo Fisher Scientific, FD0684), and insert size was verified by agarose gel electrophoresis. Correct constructs were confirmed by DNA sequencing analysis to verify seamless gene fusion and correct nucleotide sequence.

**Supplementary Tables**

**Table S1.** Key genetic elements and plasmid constructs used to generate bioluminescent *M.tb* reporter strains

| **Element** | **Description** | **Reference** |
| --- | --- | --- |
| **Parental strain** | *M.tb* H37Rv |  |
| **Promoters** |  |  |
| pL5 | Mycobacteriophage L5 promoter | [15] |
| pHsp60 | *M.tb* heat‐shock protein 60 promoter | [14] |
| pG13 | *E. coli* sigma 70-like promotor | [16] |
| **Reporter genes** |  |  |
| luxABCDE operon | Photorhabdus luminescens operon (luxAB: luciferase; luxCDE: substrate synthesis) | GenBank no. AF4430784.1 |
| frp | FMN oxidoreductase (enhances intracellular luciferin regeneration) | GenBank no. GQ850536.1 |
| **Plasmids** |  |  |
| pMV306 | Mycobacterial integrative shuttle vector; kanamycin‐resistance | [15] |
| pL5-luxAB-pG13-LuxCDE | pMV306L5+luxABCDE derivative with pG13 inserted in front of luxCDE | This study |
| pL5-luxAB-pG13-LuxCDE-frp | pL5-luxAB-pG13-LuxCDE added frp | This study |
| pHsp60-luxAB-pG13-LuxCDE | pMV306hsp+luxABCDE derivative with pG13 inserted in front of luxCDE | This study |
| pHsp60-luxAB-pG13-LuxCDE-frp | pHsp-luxAB-pG13-luxCDE added frp | This study |
| **Recombinant Strains** |  |  |
| Mtb-L5-lux | H37Rv attB:: pL5-luxAB-pG13-LuxCDE | This study |
| Mtb-L5-lux-frp | H37Rv attB:: pL5-luxAB-pG13-LuxCDE-frp | This study |
| Mtb-Hsp60-lux | H37Rv attB:: pHsp60-luxAB-pG13-LuxCDE | This study |
| Mtb-Hsp60-lux-frp | H37Rv attB:: pHsp60-luxAB-pG13-LuxCDE-frp | This study |

Note: References [1]–[45] are listed in the main manuscript.

**Table S2.** **List of primers**

| **Overlapped Extention PCR elements** | **Primer(5’-3’)** |  |
| --- | --- | --- |
|  |  |  |
| **Hsp60-lux-frp** |  |  |
|  |  |  |
| 1^st^ PCR | gcaacgcgtgcggccgcggtaca cggtgaccacaacgacgc | |
|  | gaaattgccaaacttcatagagagtcctcctgtcg | |
|  | cgacaggaggactctct atgaagtttggcaatttc | |
|  | tagttaactacgtcgacatcgat ggtacctatcaggtatat | |
|  |  |  |
| 2^nd^ PCR | tgaaatatacctgataggtaccgatcgccactagcgccgcggtcg |  |
|  | acgaaattttcttcgtcatatttgccttcctcctgg |  |
|  | ccaggaggaaggcaaat atgacgaagaaaatttcgt |  |
|  | tagttaactacgtcgacatcgat tcagctatcgaaggcttcc |  |
|  |  |  |
| 3^rd^ PCR | ggataatgaccacctggatatc gacgtggacatcaccgag |  |
|  | catttctttctccttagtcagctatcgaaggctt |  |
|  | aagccttcgatagctga ctaaggagaaagaaatg |  |
|  | cagtcgatcgtacgctagttaac tcaccgcttcgccaaacc |  |
|  |  | |
| **L5-lux-frp** |  | |
|  |  | |
| 1^st^ PCR | cgagcgcaacgcgtgcggccgc ggtaca ggaaacagctatgacctg | |
|  | aaattgccaaacttcatatgcgatctccctttcc | |
|  | ggaaagggagatcgcat atgaagtttggcaattt | |
|  | aggtaggccgccgcgacatatg ggttgccgagcaaaccga | |
|  |  | |
| 2^nd^ PCR | tgaaatatacctgataggtaccgatcgccactagcgccgcggtcg | |
|  | acgaaattttcttcgtcatatttgccttcctcctgg | |
|  | ccaggaggaaggcaaat atgacgaagaaaatttcgt | |
|  | tagttaactacgtcgacatcgat tcagctatcgaaggcttcc | |
|  |  | |
| 3^rd^ PCR | ggataatgaccacctggatatc gacgtggacatcaccgag | |
|  | catttctttctccttagtcagctatcgaaggctt | |
|  | aagccttcgatagctga ctaaggagaaagaaatg | |
|  | cagtcgatcgtacgctagttaac tcaccgcttcgccaaacc | |
|  |  | |

**Supplementary Figures**


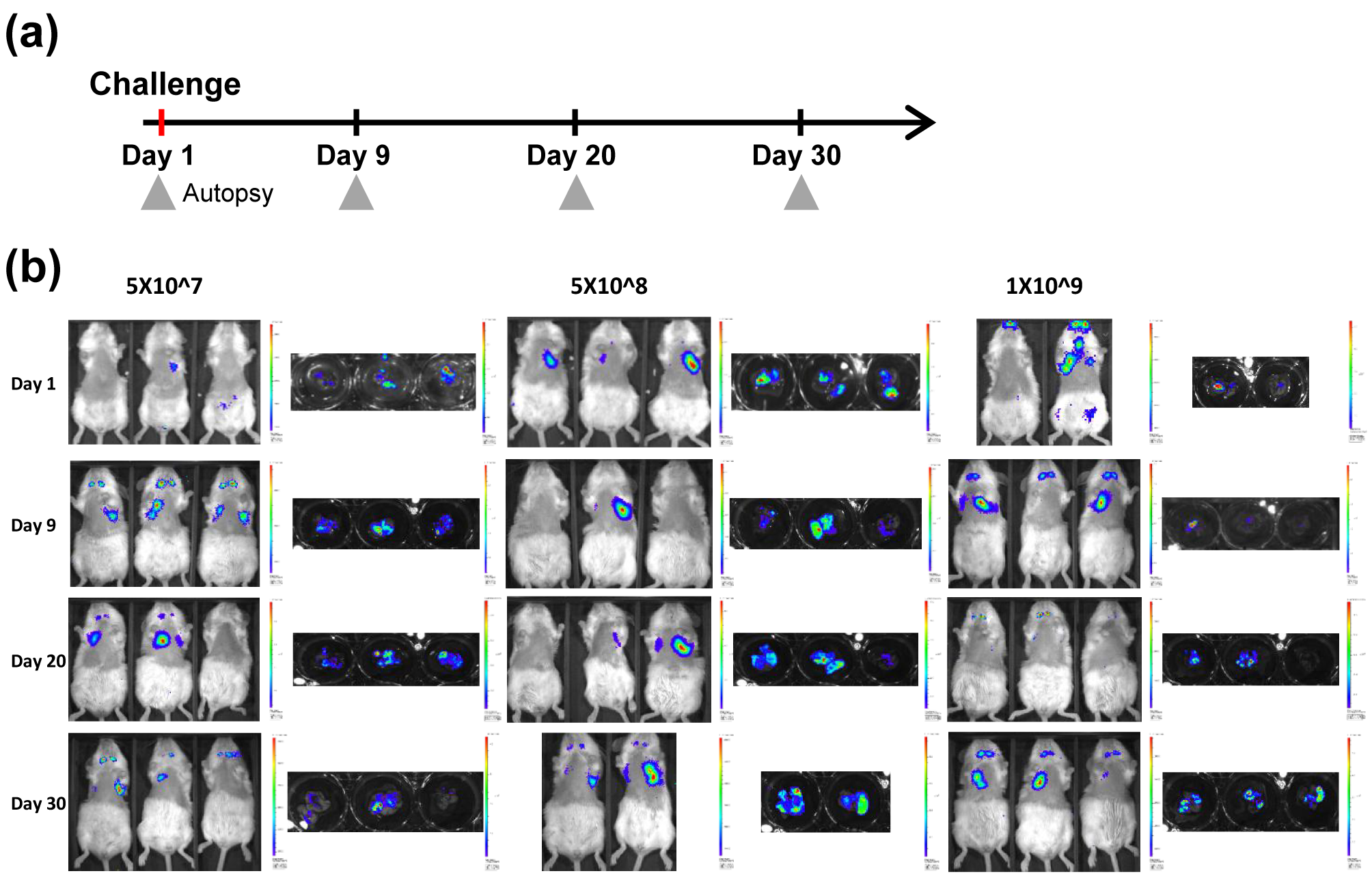


**Figure S1.** *In vivo* and *ex vivo* bioluminescence imaging to monitor *M.tb* L5 Lux infection concentration in BALB/c. (A) An experimental timeline. (B) Representative in vivo and *ex vivo* bioluminescence images of live mice at different time points post-infection and concentrations, showing the progression of *M.tb* L5 Lux infection.

**
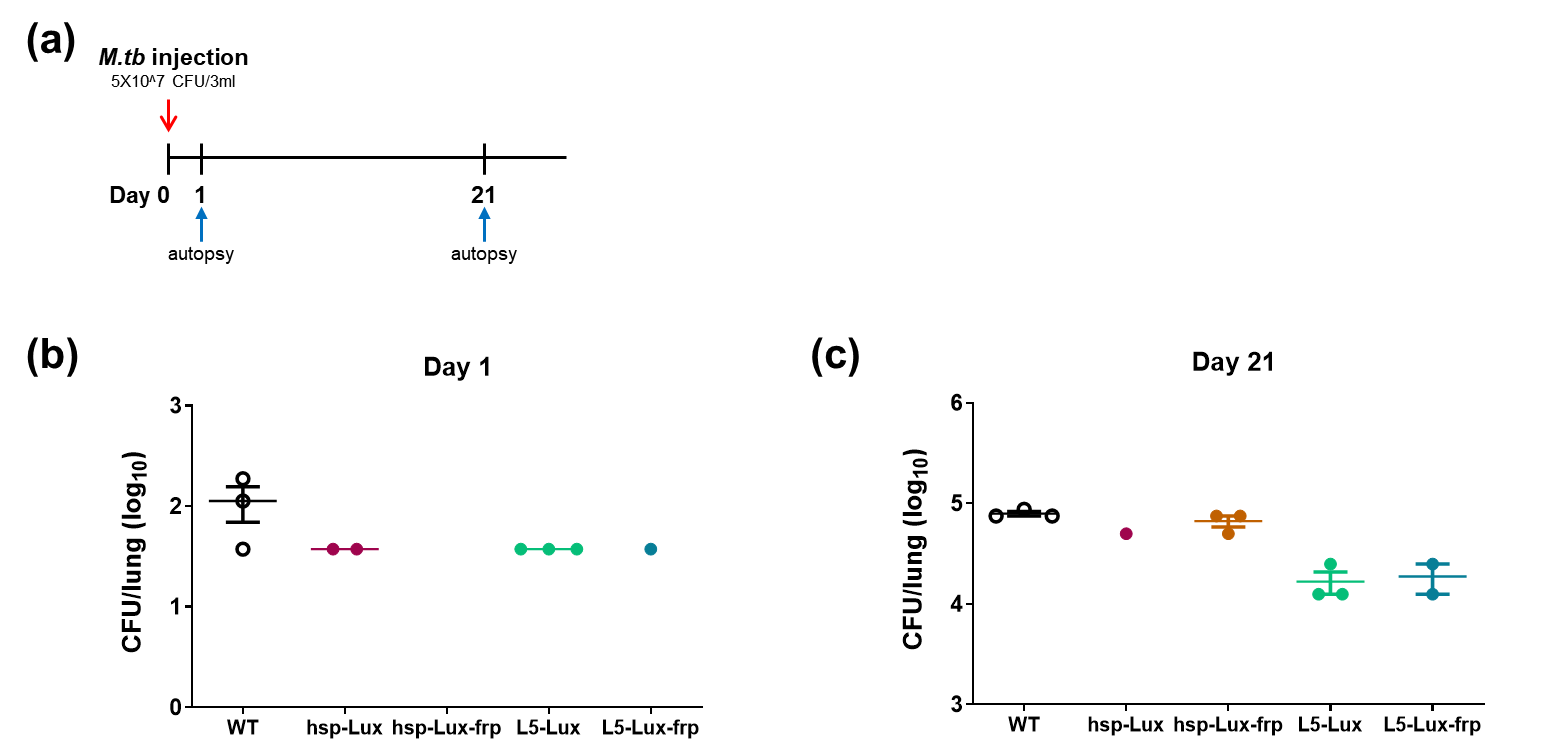
**

**Figure S2. Intranasal infection and in vivo growth of recombinant *M.tb* reporter strains.** (A) Timeline of intranasal infection and lung harvest. BALB/c mice (n = 3 per group) were infected with 5 × 10⁷ CFU of H37Rv, Hsp-Lux, Hsp-Lux-frp, L5-Lux, or L5-Lux-frp, and lungs were collected at days 1 and 21. (B) Lung CFU at days 1 and 21. Each dot represents one mouse (mean ± SD). All recombinant strains established infection, but only L5-Lux maintained lung burdens comparable to wild-type H37Rv at day 21.

#
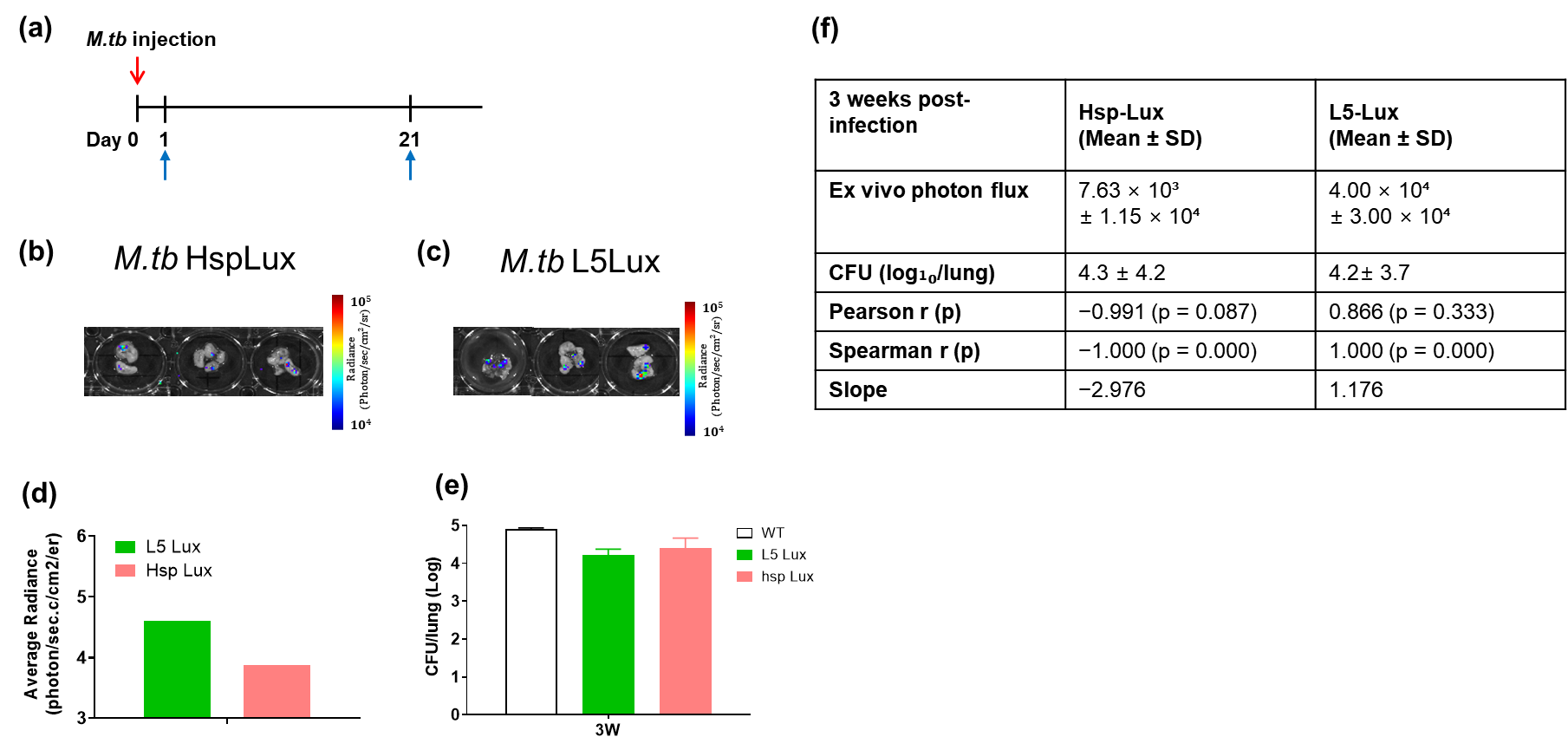
****Figure S3.** Head-to-head comparison of Hsp-Lux and L5-Lux reporter strains in an intranasal infection model.** (A) Experimental timeline. BALB/c mice (n = 3 per group) were infected intranasally with 5 × 10^7^ CFU of Hsp-Lux or L5-Lux, and lungs were imaged at day 21 post-infection. (B) Representative *ex vivo* IVIS images of lungs at day 21 (same radiance scale for both groups). (C) Lung bioluminescence (radiance, photons/s/cm²/sr). L5-Lux generated higher photon output than Hsp-Lux at comparable inocula. (D) Lung bacterial burdens (CFU) at day 21. CFU counts were similar between the two strains, indicating that the stronger signal from L5-Lux reflects higher reporter output rather than differences in bacterial load. (F) Benchmarking table summarizing the relationship between bioluminescence (radiance), CFU, and correlation parameters (slope, Pearson *r*) for Hsp-Lux and L5-Lux *in vivo*


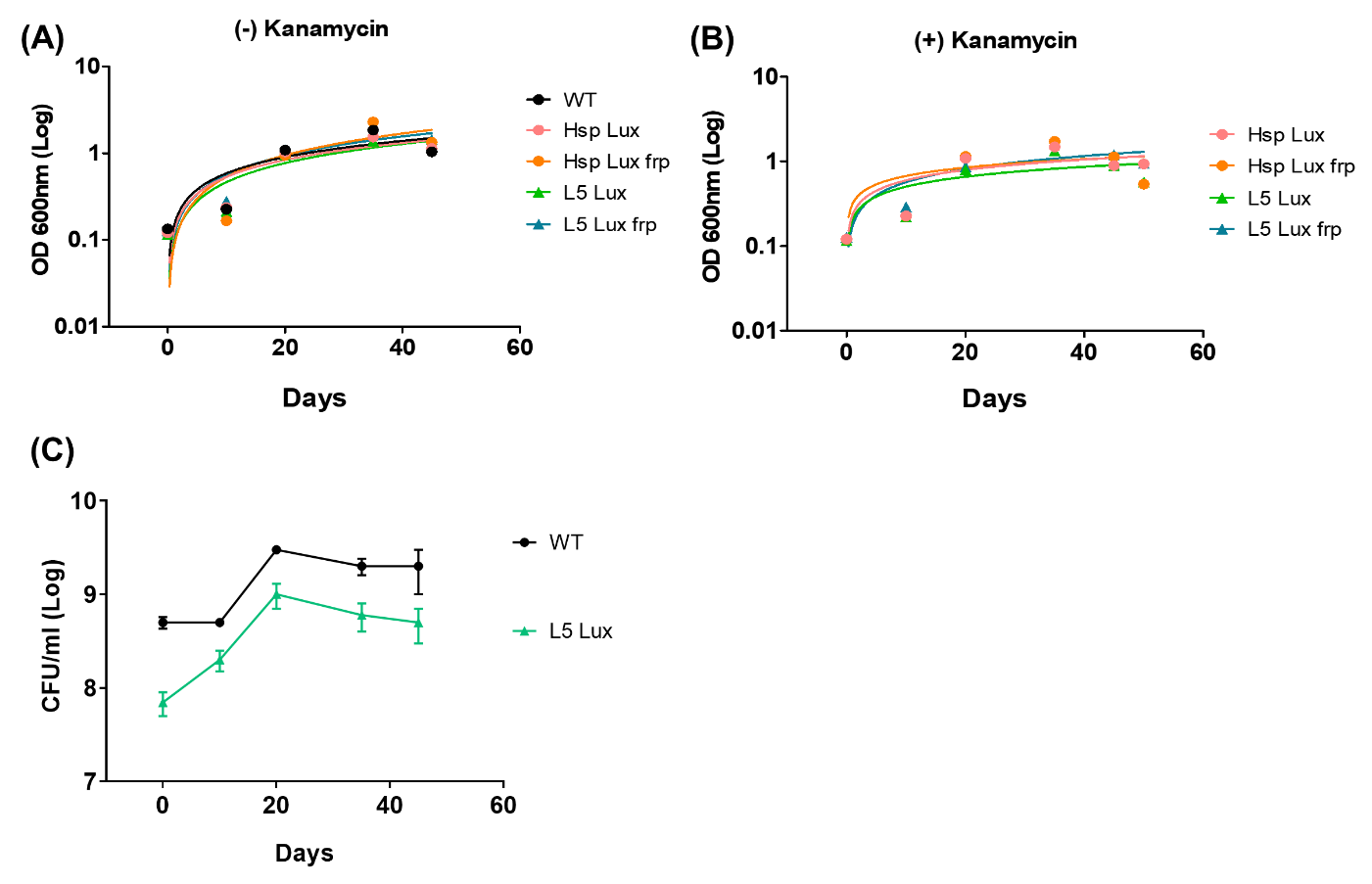
**Figure S4. Evaluation of the stability of the luminescence strains *in vitro.*** Optical density and CFU were measured to analyze the growth inhibition and stability of reporter strains containing Lux operon as reporter genes using kanamycin as a selective marker in 7H9 broth and 7H10 agar. The values represented correspond to the cultures of *M.tb* strains in (A) kanamycin-free and (B) kanamycin-added medium. (C) Wild-type and reporter strains were cultured in a kanamycin-free medium, and then CFU was counted at 7H10, including kanamycin, to confirm the stability of reporter strains.
